# Characterisation of thin films of graphene–surfactant composites produced through a novel semi-automated method

**DOI:** 10.3762/bjnano.7.19

**Published:** 2016-02-08

**Authors:** Nik J Walch, Alexei Nabok, Frank Davis, Séamus P J Higson

**Affiliations:** 1 Cranfield Biotechnology Centre, Cranfield University, College Road, Cranfield, MK43 0AL, UK; 2 Sheffield Hallam University, Materials & Engineering Research Institute, Howard Street, Sheffield, S1 1WB, UK; 3 The University of Chichester, College Lane, Chichester, West Sussex, PO19 6PE, UK

**Keywords:** characterization, ellipsometry, graphene, ^1^H NMR, surfactant

## Abstract

In this paper we detail a novel semi-automated method for the production of graphene by sonochemical exfoliation of graphite in the presence of ionic surfactants, e.g., sodium dodecyl sulfate (SDS) and cetyltrimethylammonium bromide (CTAB). The formation of individual graphene flakes was confirmed by Raman spectroscopy, while the interaction of graphene with surfactants was proven by NMR spectroscopy. The resulting graphene–surfactant composite material formed a stable suspension in water and some organic solvents, such as chloroform. Graphene thin films were then produced using Langmuir–Blodgett (LB) or electrostatic layer-by-layer (LbL) deposition techniques. The composition and morphology of the films produced was studied with SEM/EDX and AFM. The best results in terms of adhesion and surface coverage were achieved using LbL deposition of graphene(−)SDS alternated with polyethyleneimine (PEI). The optical study of graphene thin films deposited on different substrates was carried out using UV–vis absorption spectroscopy and spectroscopic ellipsometry. A particular focus was on studying graphene layers deposited on gold-coated glass using a method of total internal reflection ellipsometry (TIRE) which revealed the enhancement of the surface plasmon resonance in thin gold films by depositing graphene layers.

## Introduction

Since its initial discovery and development by Novoselov et al. [1] graphene has been of great interest to the scientific community due to its interesting optical and electrical properties. Graphene is defined as a single layer of sp^2^-hybridised carbon with no third dimension. The double-bonded structure of graphene is responsible for the electrical properties of the material as the movement of π-bonds between adjacent carbon atoms can be used to transmit an electrical current. Because of this electrical activity in particular, graphene is being examined as a base material in a number of different applications including sensor applications, for use in flexible electronics [2] and graphene-based printable inks for printed electrical circuits [3].

Graphene has reportedly been produced in a number of different ways. The method chosen for this research is sonochemical exfoliation in water in the presence of a surfactant, as first reported by Notley et al. [4]. This method was chosen for a number of reasons; firstly, it does not require the use of hazardous chemicals such as sodium nitrate, sulfuric acid, potassium permanganate and hydrazine hydrate, which are used in the oxidation of graphite to graphite oxide and the subsequent reduction to graphene [5], and secondly it guarantees single-layer or few-layer graphene, rather than the potentially larger products or graphene sheets with an uneven size distribution that might be produced in other techniques such as mechanical exfoliation (the “scotch tape” method). The sonochemical method was carried out using a semi-automated apparatus designed specifically for the purposes of this research.

In previous work [6] we manufactured graphene–surfactant complexes using the Notley method and applied them to carbon electrodes, thereby enhancing their electrochemical activity. Further work was then carried out on optimising graphene production, characterising the products and also depositing graphene layers by more controlled techniques than simple casting.

In this work a semi-automated technology of graphene production by sonochemical exfoliation of graphite in the presence of ionic surfactants is described in detail. The composite graphene-surfactant materials produced were characterised with NMR and Raman spectroscopy to confirm the formation of graphene. Thin films of graphene composites were deposited using the techniques of Langmuir-Blodgett (LB) and electrostatic layer-by-layer (LbL) deposition. Films composed of these new graphene composite materials were then characterised using SEM, AFM, and spectroscopic ellipsometry. The study of SPR in gold films coated with graphene using total internal reflection ellipsometry was carried out for the first time.

## Experimental

### Semi-automated sonochemical exfoliation of graphene

Two different surfactants were used to synthesise graphene–surfactant complexes: sodium dodecyl sulfate (SDS) and cetyltrimethylammonium bromide (CTAB). Firstly the surfactant solutions were prepared by dissolving in water. The SDS solution was made to a concentration of 462.9 mg·mL^−1^ while the CTAB solution concentration was made up to 49.7 mg·mL^−1^. These solutions were prepared and then placed into a water bath heated to 50 °C to aid dissolution.

**Figure 1:**
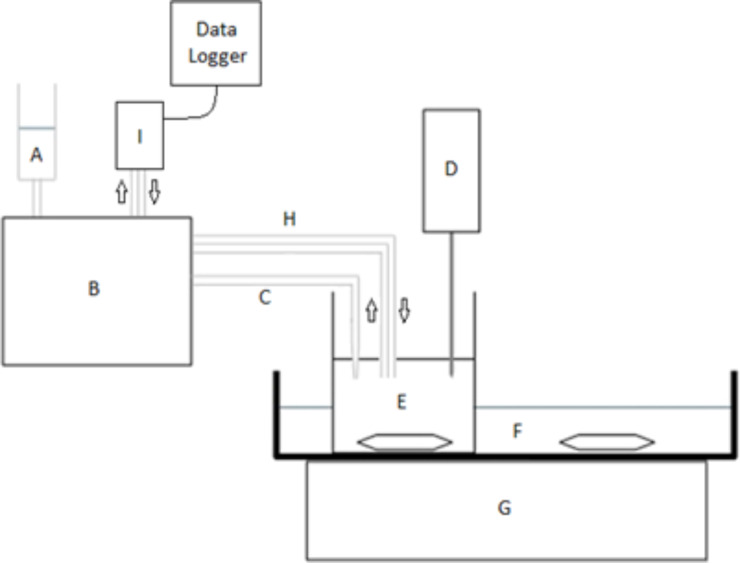
A schematic diagram of the apparatus used to synthesis graphene. (A) surfactant solution, (B) peristaltic pump, (C) heat, (D) ultrasonic probe, (E) reactor, (F) water, (G) magnetic stirring unit, (H) sampling loop, and (I) photometric flow cell.

Once dissolved, the surfactant solution was placed into the surfactant reservoir of the synthesiser. This solution was then pumped into the reactor during synthesis at a rate of 35 μL·min^−1^ giving an addition rate of 16.2 mg·min^−1^ for SDS and 1.74 mg·min^−1^ for CTAB. The addition rate was crucial to maintain a surface tension of 41 mJ·m^−2^, which is both the optimum surface tension for graphene production and also the surface free energy of graphene. The graphite suspension (10–50%) was then placed into the reactor, and sonicated continuously for 120 min at a power of 50 W. A total of 3.15 mL of surfactant solution was added in each case over the course of the synthesis. The sonication step was carried out in a fume cabinet, as an aerosol containing potentially harmful graphene nanoparticles is produced at this stage. A schematic diagram of this apparatus is shown below (Figure 1).

The apparatus was designed to alleviate the time constraints of the Notley method. In that method, solid surfactant must be added every 5 min whilst continuous sonication is carried out. This method with its continuous addition of aqueous surfactant eliminates the need for constant supervision of the process as well as providing the reproducibility of automation. This is only semi-automated, however, as full automation would require the monitoring of surface tension throughout the synthesis as well as automatic adjustments to the surfactant flow rate to maintain this at the required level of 41 mJ·m^−2^.

Figure 1 depicts the assembled apparatus, the function of which is explained here. The aqueous suspension of graphite is placed into the reactor (E). The graphite is kept in suspension throughout the procedure using a magnetic stirrer (G). The ultrasonic probe (D), operating at a power of 50 W, penetrates approximately 1 cm into the graphite suspension. The surfactant solution (A) is pumped into the reactor via a peristaltic pump (B). The reservoir containing the surfactant and the tubing carrying it to the reactor are heated to 50 °C in a water bath (C) to prevent precipitation of the surfactant from solution in the tubing. A water trough (F) is used as a heat sink to dissipate any heat produced in the reactor during synthesis, as the tip of the ultrasonic probe can reach temperatures of up to 100 °C during continuous operation. The heat sink is stirred continuously, also using the magnetic stirrer (G), to maximise cooling efficiency. A sampling loop (H) continuously cycles the liquid contents of the reactor through a photometric flow cell (I) which determines the concentration of graphene by measuring the absorbance at 650 nm. The concentration data is then recorded using a data logger.

Upon completion of the 120 min cycle, the contents of the reactor were removed and placed into a 50 mL centrifuge tube and spun at 3500 rpm for 20 min. The supernatant was then removed and the pellet (containing mostly unreacted graphite as well as some precipitated graphene) was re-suspended in 40 mL of deionised water. This was then centrifuged at 3500 rpm for 20 min. This process was repeated until no more graphene was contained in the supernatant. If a dry sample was required, then the graphene suspension was frozen and then placed into a freeze dryer (Labogene) for 3–4 days (depending on the sample size) and then subsequently into a vacuum oven at 70 °C overnight to remove all traces of water.

### Characterisation of graphene-surfactant composite material

Graphene–surfactant complexes were characterised by using a range of different techniques. The presence of graphene was determined by Raman microscopy. The concentration of graphene in solution was obtained by spectrophotometry. The absorbance of a graphene solution at a wavelength of 650 nm was determined and the Beer–Lambert law applied, using an extinction coefficient of 13.9 mg·mL^−1^·cm^−1^ [7]. The interaction of the surfactant with the graphene surface was also analysed using NMR spectroscopy. Dried graphene–surfactant complex was dissolved in D_2_O and placed into an NMR tube. Another tube containing the surfactant alone was also prepared. Both were scanned individually and then the spectra were compared to show shifts in peak position corresponding to interaction between the surfactant and the graphene. NMR experiments were performed on a Bruker 400 MHz spectrometer running topspin analysis software. This analysis works according to the nuclear Overhauser effect (NOE). The NOE, which is present in nuclear magnetic resonance (NMR) spectroscopy, can be used to determine the amount of contact between the surfactant and the graphene sheet. This is achieved by observing peak shifts that occur when two nuclei are within 5 Å of each other. The dried graphene sample was deposited onto a glass microscope slide and then scanned using a Renishaw In-Via Raman microscope at an excitation wavelength of 532 nm. Data were recorded between 100 and 3200 cm^−1^.

### Deposition of thin films of graphene-surfactant composites

#### Langmuir–Blodgett and Langmuir–Schaefer deposition

Both composite materials obtained, e.g., graphene(+)CTAB and graphene(−)SDS, appeared to be soluble in water due to the presence of ionic groups, NMe_3_^+^ and SO_3_^−^, respectively. Yet, the presence of alkyl chains and π-systems facilitated their solubility in chloroform, hence the use of the Langmuir–Blodgett (LB) technique (Nima 610 trough) was an obvious choice for the deposition of thin films. The standard LB procedure was applied [8]: a 1 mg·mL^−1^ solution of graphene(+)CTAB in chloroform was spread onto the surface of deionised water (Millipore). Surface pressure was then recorded using a Wilhelmy plate-based sensor. Because of the unknown ratio of graphene/surfactant within the complex the area per molecule (or repeated unit) was difficult to calculate, so the area was presented in actual units of cm^2^. Another method known as Langmuir–Schaefer (LS) deposition [9] was also used to prepare monolayer films. In this technique the hydrophilic substrate is held horizontally to the assembled monolayer and then lowered slowly to gently touch water surface and the monolayer is then transferred onto the substrate surface. Organised monolayer films obtained in this fashion were then characterised by AFM (Nanoscope III) operating in tapping mode using Veeco cantilevers with silicon nitride tips having a radius of less than 10 nm.

#### Electrostatic LbL deposition

Much better results (in terms of adhesion and surface coverage) were obtained by using a simple technique of electrostatic layer-by-layer (LbL) deposition, a well-established technique developed first for polyelectrolytes [10] and later adapted for deposition of other objects, such as nanoparticles and biomolecules (proteins, antibodies, enzymes, DNA) [9,11]. Multi layered films of graphene were deposited onto gold-coated glass microscope slides by alternating layers of graphene–surfactant with oppositely charged polyelectrolytes, e.g., graphene(+)CTAB layers alternated with polyanionic layers of sodium polystyrene sulfonate (PSS), while graphene(−)SDS was alternated with layers of polycationic species such as polyallylamine hydrochloride (PAH) or polyethyleneimine (PEI). Alternating layers of graphene(−)SDS and graphene(+)CTAB was also attempted. The films were deposited by consecutive dipping of gold-coated glass or silicon wafers into 1 mg·mL^−1^ solutions of the above materials in deionised water. The mutlilayered films obtained were then characterised with scanning SEM combined with EDX (energy dispersing X-ray) elemental analysis (SEM NOVA) and AFM.

### Optical characterisation of thin graphene-surfactant films

#### Spectroscopic ellipsometry study

UV–vis spectra of graphene–surfactant samples are featureless showing almost constant absorbance over the spectral range of 350–800 nm, while the main absorption band of graphene lies in the UV region at about 280 nm. Therefore, optical properties of novel graphene–surfactant composites were studied via spectroscopic ellipsometry using a J. A. Woollam M2000 instrument operating in the spectral range of 370–1000 nm. The measurements were performed on graphene–surfactant films deposited on different substrates, i.e., glass, silicon, and gold-coated glass. Experimental parameters were found by fitting data, the procedure for which is explained in the Results and Discussion section below.

For the LbL deposition of alternating layers of graphene(−)SDS and graphene(+)CTAB on gold-coated glass slides, a gold film of approximately 25 nm thickness was thermally evaporated onto a 3 nm under layer of chromium (which was used to improve adhesion between the gold and the glass). Metal evaporation was carried out in a vacuum of 10^−6^ Torr using an Edwards 360 unit. Prior to LbL deposition, gold-coated slides were treated overnight in cystamine hydrochloride solution in order to make the gold surface positively charged. Then LbL deposition started with the layer of graphene(−)SDS (negatively charged) followed by deposition of graphene(+)CTAB (positively charged). This procedure was repeated four times, so that four graphene bilayers were deposited. Layer by layer deposition onto other substrates was performed in a similar manner.

#### TIRE study

The surface plasmon resonance (SPR) phenomenon in graphene films deposited on thin films of gold was studied in more detail using the method of total internal reflection ellipsometry (TIRE), which was developed in the last decade [12–14]. TIRE experimental set-up (shown schematically below as inset in Figure 12) was built on the basis of a J. A. Woollam M2000 spectroscopic instrument, in which the light is coupled into a thin gold film deposited on glass through a 68° prism providing total internal reflection conditions. The cell attached underneath allows for measurements in different media. The advantage of using TIRE is a 10-fold sensitivity enhancement compared to traditional SPR [15]. The samples were constructed by electrostatic LbL deposition of PEI and graphene(−)SDS on chromium/gold-coated glass slides, as described above.

## Results and Discussion

### Characterisation of graphene–surfactant composite material

**Figure 2:**
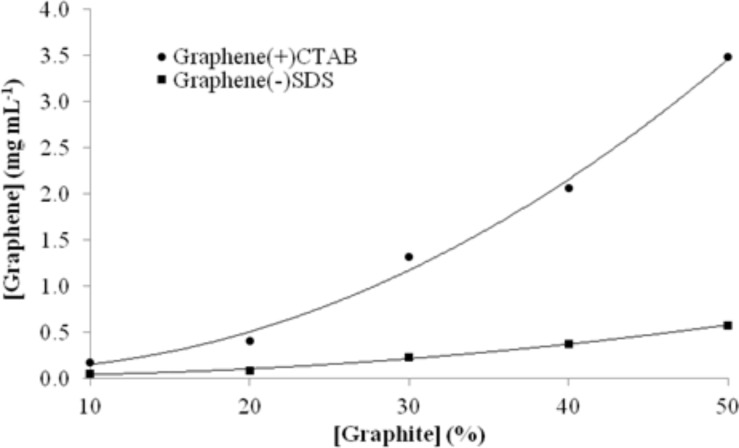
Effect of the initial graphite suspension concentration in water on the final graphene concentration after sonication for 120 min in the presence of different surfactants.

Graphene was synthesised using graphite suspensions of 10–50% using either SDS or CTAB as the surfactants. The concentration of the final graphene solution obtained from each synthesis was determined by spectrophotometry (Figure 2).

**Figure 3:**
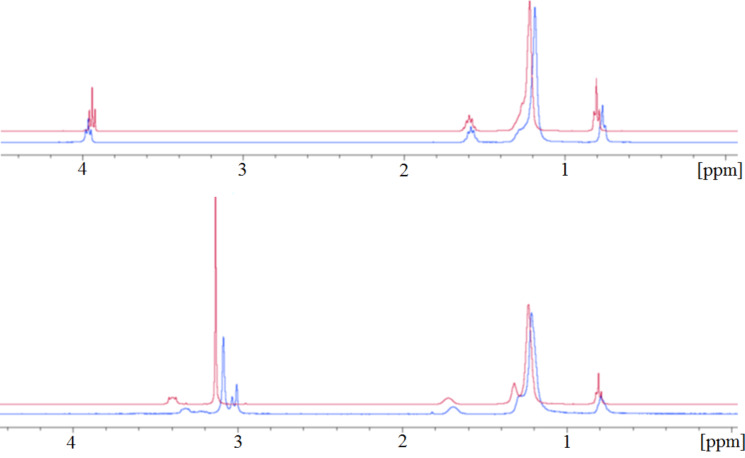
^1^H NMR spectra of the graphene–surfactant complexes (blue) stabilized with SDS (top) and CTAB (bottom) compared with the surfactant alone (red).

^1^H NMR measurements of the graphene–surfactant complex, when compared to ^1^H NMR measurements of the surfactant alone, shows shifting of peaks representing hydrogens involved in the complexation interaction (Figure 3). The data shows a peak shift towards the left for almost every peak. This is a shielding effect caused by the delocalised electrons in the graphene sheet, which only occurs when the proton is in close proximity (less than 5 Å) and involved in van der Waals interactions. This suggests that the hydrophobic chains of each surfactant lie flat against the graphene sheet with the exception of carbon-1 (the carbon attached directly to the polar head group), which is pulled away from it by the polar head group and therefore does not undergo as much of a shielding effect in the SDS–graphene complex.

Additionally, for the CTAB–graphene complex, two of the methyl groups on the quaternary amine are interacting with the graphene sheet. This is shown by the splitting of the peak into three peaks. This interaction causes the CTAB to lie much flatter against the graphene than SDS, resulting in the polar head being pulled closer to the sheet. This is why the peak for carbon-1 in the CTAB–graphene complex is displaced further towards the left than its equivalent in the SDS-stabilised graphene.

The peak representing carbon-1 in SDS (at around 4 ppm) is shifted downfield by complexation with the graphene. This means that the protons are deshielded by the presence of graphene. Since the carbon–sulfur bond is polar the electron density around the carbon atom is already lower than it would normally be in a carbon–carbon bond. Repulsion between the graphene and the sulfate group could cause lengthening of the carbon–sulfur bond. This could in turn lead to a lower electron density around the nuclei responsible for this peak [16].

**Figure 4:**
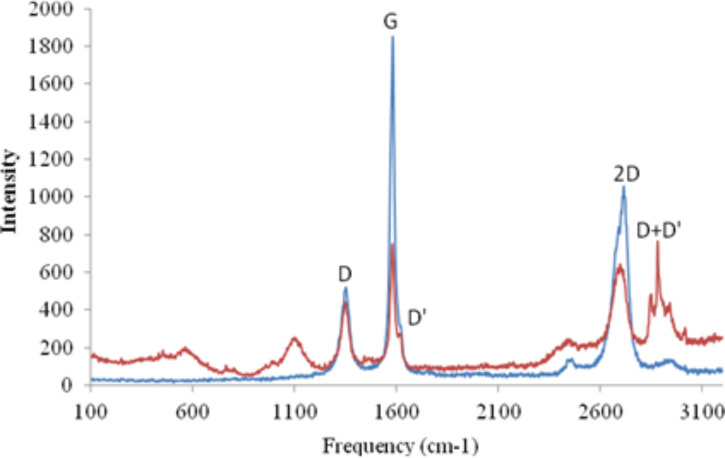
The Raman spectrum of surfactant-stabilised graphene (red) compared with the spectrum of graphite (blue).

The sample was also analysed using Raman spectroscopy (Figure 4) which, when compared to the spectrum for graphite, was used to verify the presence of graphene. The spectrum shows intense peaks at 1350 cm^−1^ (D) and 1620 cm^−1^ (G & D’). Additionally the peak labelled 2D is slightly broader, between 2650–2700 cm^−1^. This is indicative of graphene flakes with a high number of edge defects [17].

The G band is present in all sp^2^-hybridised carbon materials, and is caused by stretching of the C–C bond. The reduction in intensity in the graphene spectrum compared with the graphite spectrum is caused by the reduced number of layers. The D and D’ bands are caused by disorder in the graphene flakes. The D’ band is present when there are surface defects, such as charging or other impurities adsorbed onto the surface. The D band is caused by edge defects such as a “zig-zag” or “chair” shape on the edge. Edge defects provide an enhancement to electrochemical systems by increasing the total capacitance of the electrode surface. Both the D and the D’ band are not present in pristine graphene with straight edges [17].

The 2D band is also present in many sp^2^-hybridized systems and can be used to estimate the number of layers [18]. However the intensity is also dependant on the excitation laser frequency and so cannot be solely relied upon. Further details on the electrochemistry and usage of graphene produced by this method are detailed in another paper [6].

### Deposition of thin films of graphene–surfactant composites

#### Langmuir–Blodgett (Langmuir–Schaefer) deposition

**Figure 5:**
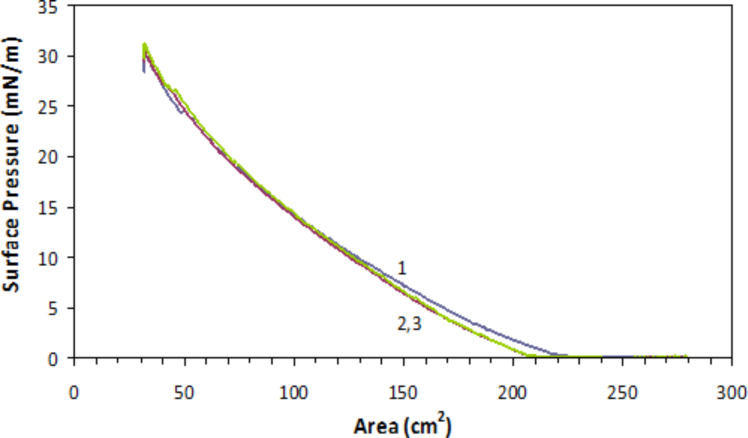
Surface pressure/area isotherms of graphene(+)CTAB monolayers on the water surface. Numbers 1, 2, and 3 indicate consecutive compressions of the monolayer.

Typical surface pressure vs area diagrams of graphene(+)CTAB in Figure 5 showed the formation of a stable monolayer on the water surface, similar to that found for classical amphiphilic compounds. The consecutive compressions of the monolayer did not yield substantial losses of material caused either by the monolayer collapse or dissolving the material in water. Graphene(−)SDS composite showed a similar behaviour.

However, for LB deposition onto hydrophilic substrates such as glass or oxidised silicon, which are slightly negatively charged due the presence of OH^-^ groups, positively charged graphene(+)CTAB was chosen; as this provided good adhesion between the substrate and monolayer.

Attempts at depositing thin films of graphene(+)CTAB using a traditional LB process, e.g., vertical dipping and withdrawing the substrate through the monolayer, were not successful since the transfer ratio was poor. The first withdrawal yielded about 60%. After that the layer was shown to peel off during subsequent dipping cycles. The overall transfer ratio by area (when substrate surface area was compared with graphene LB isotherm) was 10–20%. The most significant cause of this was poor adhesion of the first graphene layer to hydrophilic substrates. This could be improved in future work through the use of substrates with surface modifications that either enhance the surface charge or make the surface more hydrophobic. Much better results were obtained using the horizontal lifting method known as Langmuir–Shaefer (LS) deposition [8], in which the hydrophilic substrate is held horizontally to the assembled monolayer and then lowered slowly to gently touch the water surface. The monolayer is then transferred onto the substrate surface. Only a single layer of graphene(+)CTAB could be deposited by LS deposition. Attempts to deposit multilayers by the LS technique failed, as the deposited layers began to peel off upon consecutive depositions.

**Figure 6:**
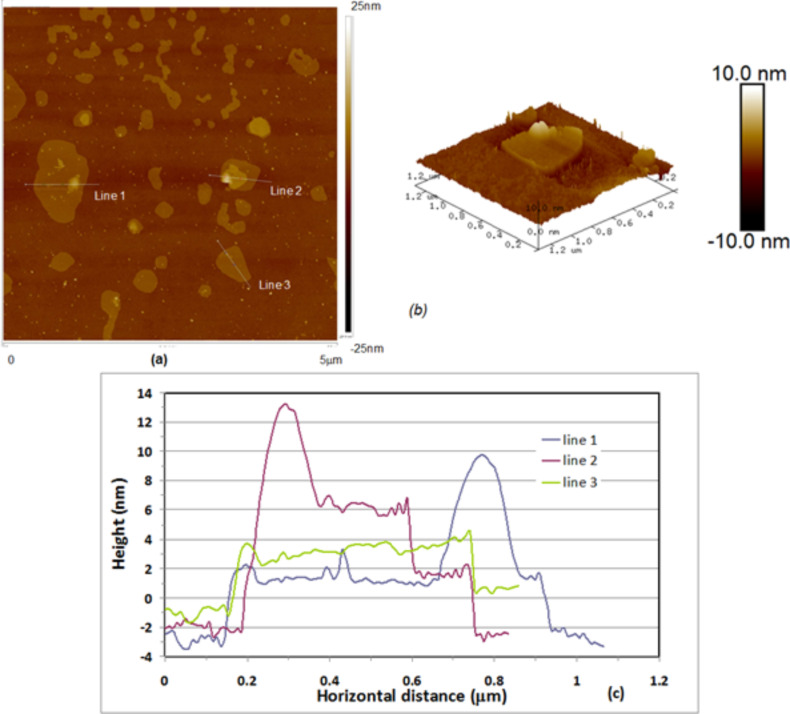
AFM images of the same sample of a graphene(+)CTAB layer deposited onto a silicon substrate using the LS method: (a) 2D image 5 μm; (b) pseudo-3D image of individual graphene flake. (c) Sectional analysis of the image in (a).

Organised monolayer films obtained in this fashion were then characterised by using AFM (Nanoscope III) operating in tapping mode using Veeco cantilevers with silicon nitride tips having a radius of less than 10 nm. A typical AFM image of graphene(+)CTAB flakes deposited onto a piece of silicon wafer using LS method is shown in Figure 6. The larger scale image (a) shows a number of irregularly shaped graphene(+)CTAB flakes with gaps between; the flakes were sometimes shown to overlap, forming double and sometimes triple layers. Image (b) shows, a pseudo 3D image of a flat individual flake of about 500 nm in size with another smaller flake lying on top.

Sectional analysis of AFM image along the lines shown, allows for an estimation of the graphene(+)CTAB flake thickness at 2 nm, which is significantly higher than the reported thickness of pristine graphene of 0.355 nm [19]. The obtained value of 2 nm for an individual graphene(+)CTAB is likely due to the presence of surfactant molecules, CTAB in this case.

It is clear from these images that the surface coverage is not optimal. Additionally the graphene flakes were seen to change position and shape during scanning, suggesting poor adhesion to the silicon surface. This could potentially be overcome in future work by using surface-modified silicon wafers for sample deposition.

#### Electrostatic LbL deposition

The multi layered films obtained from layer-by-layer deposition method were characterised with scanning SEM combined with EDX (energy dispersing X-ray) elemental analysis (SEM NOVA) and AFM.

Not all alternating combinations worked well, however. For example, the most promising combination of graphene(+)CTAB with graphene(−)SDS was not successful, while the alternation of graphene(+)CTAB with PAH (or with PEI) proved to be optimal. Deposition on glass or silicon samples was performed by electrostatic adsorption of PAH (or PEI) for 20–30 min followed by dipping into a solution of graphene(−)SDS for 10–15 min. This sequence was repeated several times with a typical incubation time of 10–15 min.

**Figure 7:**
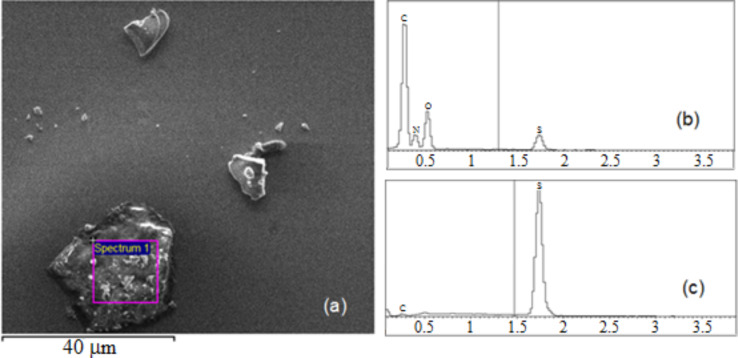
(a) SEM image of PAH/graphene(−)SDS layer on a silicon surface; (b) EDX spectra recorded on a graphene flake, and (c) an empty space.

Figure 7 shows an SEM image of alternating layers of PAH and graphene(−)SDS deposited onto a silicon substrate. Separate flakes are clearly visible, the largest of which is approximately 30 μm across. EDX spectral analysis (b) performed on a flake show a dominating peak of carbon while on the empty space (c) silicon is the dominant peak. This shows that the graphene flakes consist predominantly of carbon, with a few trace elements. Deposition of the first few layers gives a less than optimal coverage. The reason for this is likely poor adhesion between layers of graphene(−)SDS and PAH. Deposition of subsequent layers greatly improves the coverage by overlapping adjacent graphene flakes.

**Figure 8:**
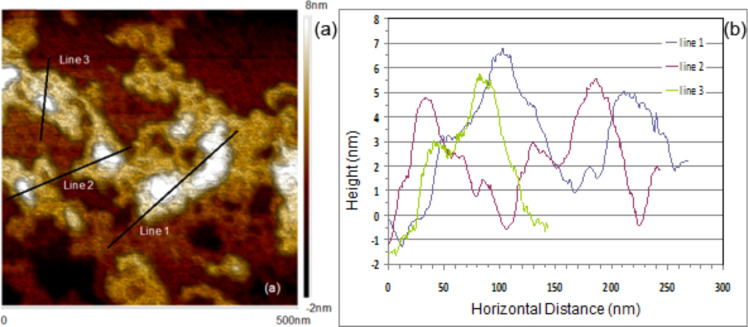
(a) AFM image (tapping mode) of a PEI/graphene(−)SDS film, and (b) a corresponding sectional analysis.

Adhesion between graphene and substrate was greatly improved when using a branched polycation such as PEI. The AFM image of graphene(−)SDS deposited onto a layer of PEI in Figure 8 shows far better surface coverage. However it can be seen that graphene flakes overlap and form double and, in some cases, triple layers.

Sectional analysis performed on the sample shows the double and triple layers, and shows the thickness of a single flake at approximately 2.5 nm. This is a somewhat higher than that for LS films in Figure 6; the difference could potentially be caused by the presence of the PEI layer.

### Optical characterisation of thin graphene-surfactant films

#### Spectroscopic ellipsometry study

**Figure 9:**
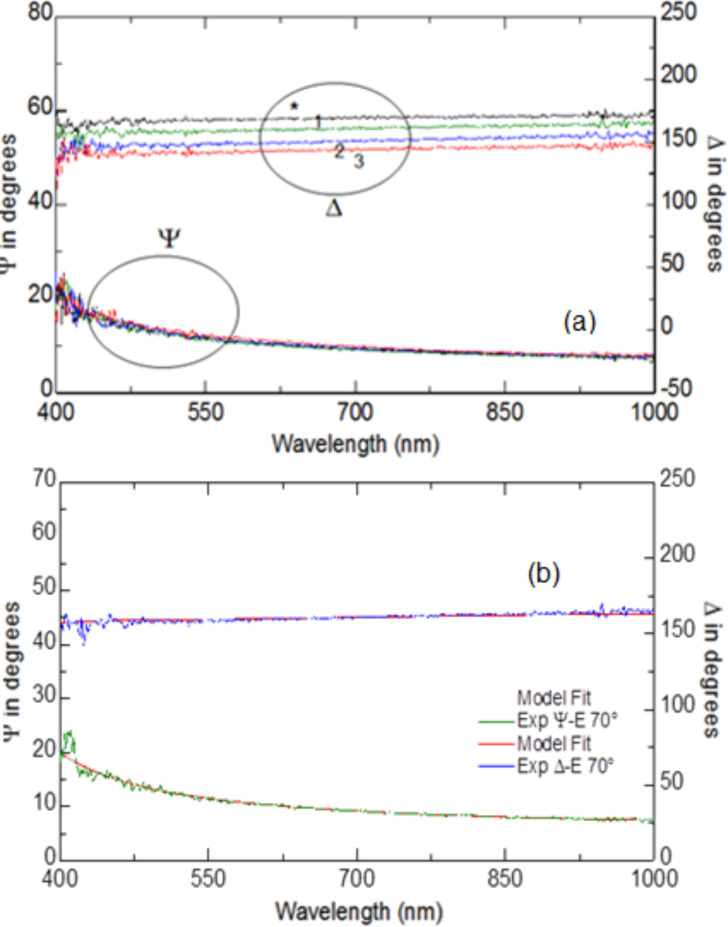
(a) Spectra of ellipsometric parameters Ψ and Δ recorded on a bare silicon surface (*) and on PAH/graphene(−)SDS films deposited on it (the numbers 1, 2, 3 correspond to the number of PAH/graphene bilayers); (b) The example of data fitting for one bilayer of PAH/graphene deposited on silicon using a three-layer model.

The analysis of graphene films by spectroscopic ellipsometry has already been carried out by other groups, and so is fairly well described [20–21]. A typical set of spectra of Ψ and Δ of graphene(−)SDS deposited on Si by alternation with PAH are shown in Figure 9a. Numbers 1, 2, and 3 indicate the number of PAH/graphene bilayers deposited. It can be seen from the data that all Ψ spectra almost coincide with each other, while the Δ spectra shift downwards upon deposition of bilayers of PAH/graphene(−)SDS.

The thickness values (*d*) and dispersions, e.g., spectra of refractive index (*n*) and extinction coefficient (*k*) of graphene films can be found by fitting the above spectra to the model using the dedicated software by J. A. Woollam. In this particular case, the model of the reflecting system consists of the following three layers: (1) Si substrate; (2) the layer of native oxide (SiO_2_) which is typically present on the surface of Si; (3) the deposited layer of PAH/graphene(−)SDS. The ambient was air. Optical parameters for Si and SiO_2_ were taken from the J. A. Woollam database. The fitting was first performed for ellipsometric spectra of the Si/SiO_2_ substrate. For the set of data in Figure 9a, the fitting for the thickness of the native SiO_2_ layer was performed first using the data for the bare Si substrate. The thickness of SiO_2_ layer obtained (*d* = 3.2 nm) was then fixed for consecutive fittings. The PAH/graphene(−)SDS film was considered as one layer in the following fittings which is justified by the fact that aliphatic chains of SDS interlock with PAH and form a mixed composite layer of PAH/graphene(−)SDS. Several possible models were tried for fitting PAH/graphene(−)SDS layers, and the best results were achieved using a Lorentz oscillator model from the J. A. Woollam data analysis software, which is given below as a dispersion function of a complex dielectric permittivity, ε(hν):


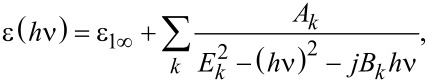


where ε_1∞_ is the dielectric permittivity at infinite frequency, *E_k_*, *A_k_* and *B_k_* are, respectively, the position, amplitude, and half-width of the *k*-th Lorentzian peak. There could be a number of peaks from 1 to *k*. The best fit was obtained with the use of a single Lorentzian with the following parameters: ε_1∞_ = 1.31, *E_k_* = 0.625 eV, *A_k_* = 1.759 (eV)^2^, *B_k_* = 3.86 eV. The presence of the Lorentz peak in the IR region gives a featureless dispersion of *k* for graphene–surfactant composite films in the visible spectral range similar to that reported in [22–23]. The absorption peak of graphene reported earlier [22–23] at about 260 nm is outside the spectral range of our ellipsometric instrument (370–1000 nm). The example of ellipsometry data fitting for one PAH/graphene bilayer deposited on Si is shown in Figure 9b with the dotted (fitting) lines almost perfectly reproducing the experimental spectra (solid lines). The thicknesses were found to be of 6.65, 9.3, and 10.88 nm for 1st, 2nd, and 3rd PAH/graphene bilayer, respectively. Although the *d* value of the first bilayer appeared to be too high, the average thickness increment Δ*d* = 3.63 ± 2 nm is reasonable and close to that observed with AFM.

**Figure 10:**
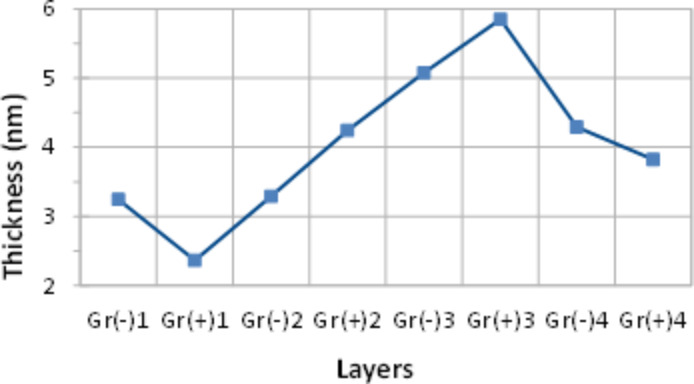
The variation of the film thickness upon deposition of alternating graphene(−)SDS and graphene(+)CTAB graphene layers on gold-coated glass slides.

Spectroscopic ellipsometry measurements were carried out on samples after each layer was deposited and similarly to the previous experiments, Ψ spectra did not change much while the Δ spectra exhibited downward shifts upon deposition of the layers. The ellipsometry data fitting was performed in a similar way as described above using a three layer model containing the substrate glass, the chromium/gold layer, and the graphene layer. The ambient was air. The parameters *d*, *n*(λ) and *k*(λ) of the chromium/gold layer were found by fitting the data for uncoated samples, and then used as fixed parameters for subsequent fittings. The graphene layers were modeled through a Lorentz oscillator as before, the values for thickness obtained are plotted against the graphene layers deposited in Figure 10. As one can see the deposition is not consistent, the graphene layers started to peel off after 3rd deposition most likely because of poor adhesion between graphene layers. However, the thickness increment of 0.87 ± 0.03 nm in the middle of the graph is much smaller and corresponds to graphene–surfactant layers without intermediate polycation layers.

**Figure 11:**
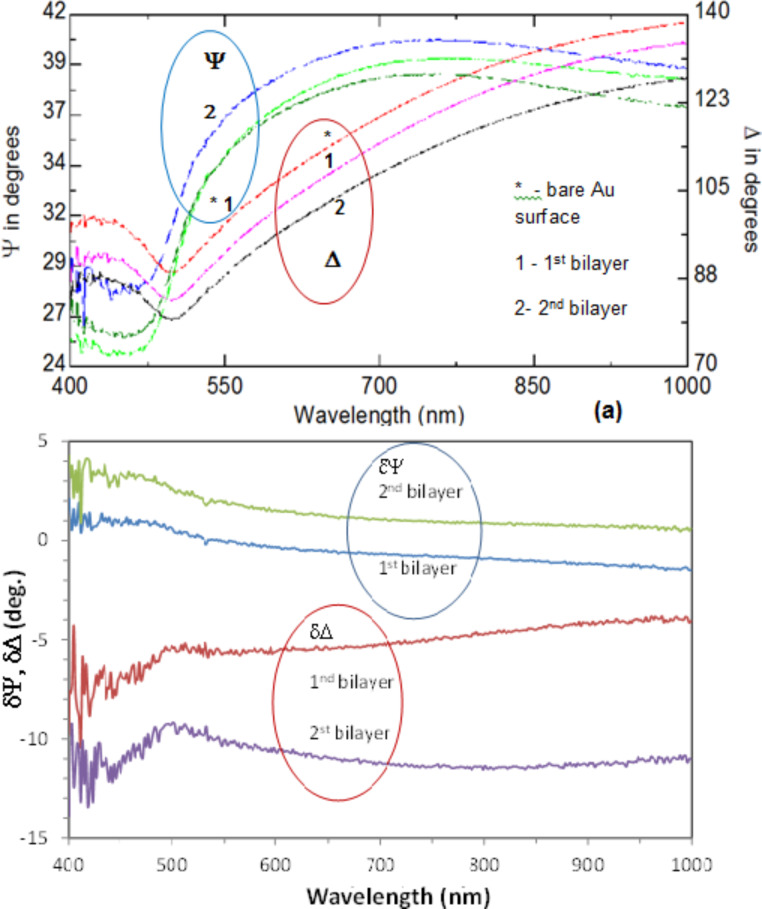
(a) Ψ, Δ and (b) δΨ, δΔ spectra of PEI/graphene(−)SDS films deposited on gold-coated glass slides.

As mentioned above, the use of PEI as a binding layer alternating with graphene(−)SDS improving the deposition of graphene. The ellipsometry spectra recorded on samples of PEI/graphene(−)SDS deposited on gold-coated glass are shown in Figure 11a. Both Ψ and Δ spectra show the characteristic features at around 450 nm associated with surface plasmon oscillations in thin gold films. Also, the spectra shift upwards and downwards, respectively, upon deposition of PEI/graphene layers, which is consistent with the thickness increment of 2.5 nm obtained by fitting of the data in Figure 11a. Following the approach developed in [24], in Figure 11b we attempted to present these data as differential spectra of δΨ = Ψ – Ψ*, and δΔ = Δ – Δ* (Ψ* and Δ* correspond to spectra of uncoated gold samples), which allows one to clearly distinguish a contribution of deposited layers.

It is quite remarkable to observe spectral features around the plasmon-resonance frequency of the gold substrate, which are not related to graphene itself [22–23] but rather appeared as a result of the interaction of π-electrons in graphene with free electrons in the gold film.

#### TIRE study

**Figure 12:**
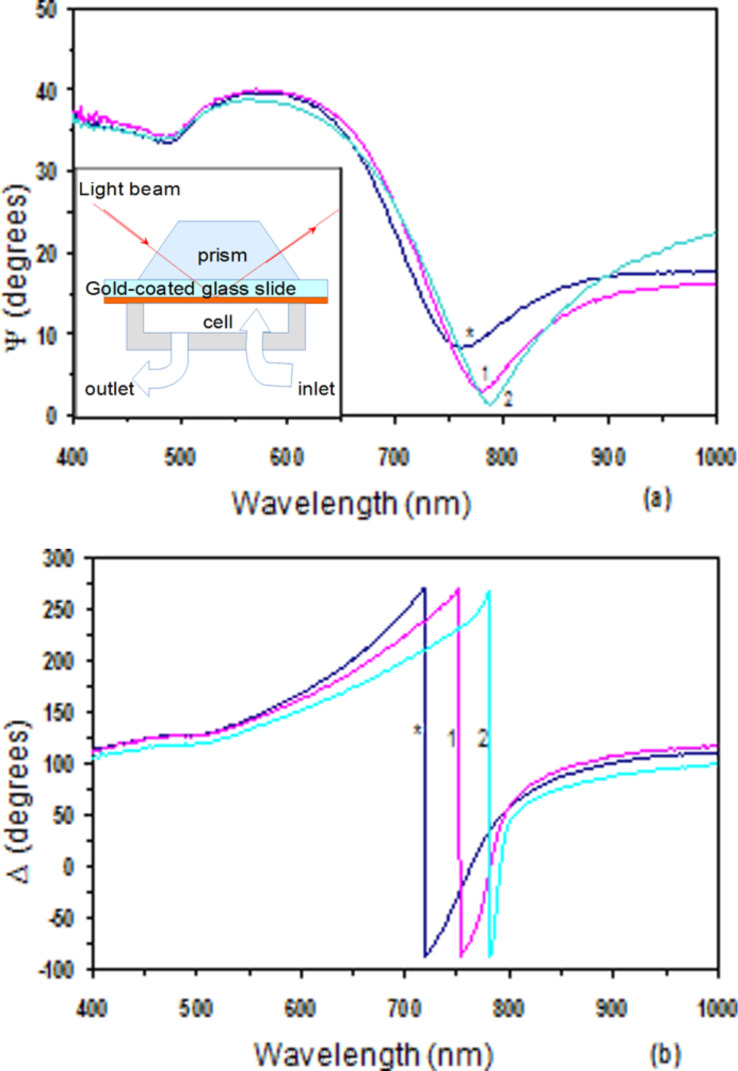
TIRE spectra of (a) Ψ, and (b) Δ recorded on a bare gold-coated glass slide (*) and after deposition of bilayers of PEI/graphene(−)SDS. Numbers 1, 2, and 3 indicate the number of bilayers deposited. The inset shows the geometry of TIRE set-up.

The samples were constructed by electrostatic LbL deposition of PEI and graphene(−)SDS on chromium/gold-coated glass slides, as described above. The results obtained are shown in Figure 12 as TIRE spectra of Ψ and Δ. The spectra of Ψ resemble a traditional SPR curve with the maximum corresponding to conditions of total internal reflection of light, while the minimum is the actual plasmon resonance. The spectra of Δ, which do not exist in traditional SPR, represent a new phase-related characteristic and show a sharp drop near the resonance wavelength.

Deposition of graphene layers made the SPR minimum even lower on the Ψ spectra while causing an additional phase shift on Δ spectra. The enhancement of the SPR peak due to the deposition of graphene layers is observed for the first time. It is interesting to note that graphene itself does not show plasmonic behavior in the visible spectral range as was proven by ellipsometry measurements shown before (Figure 9). At the same time the interaction of π-electrons in graphene and free electrons in gold (which we suspected earlier) may lead to the enhancement of SPR in gold layers. The TIRE data fitting revealed a similar thickness increment of 2.5 nm per PEI/graphene bilayer which was reported earlier.

## Conclusion

A simple semi-automated technique for graphene production by aqueous sonochemical exfoliation of graphite in the presence of ionic surfactants, e.g., CTAB or SDS, was developed. Full automation could be potentially achieved by adding surface tension sensors to control the amount of surfactant being added to the reactor, thus maintaining a constant and optimum surface tension. The formation of individual graphene flakes and the interaction of alkyl chains of the surfactants with graphene were, respectively, confirmed with Raman spectroscopy and NMR measurements.

The two different graphene-surfactant complexes produced (graphene(+)CTAB and graphene(−)SDS) appeared to be soluble in water and thus suitable for electrostatic LbL deposition. Both compounds were also found to be amphiphilic and soluble in chloroform, hence it was possible to form stable monolayers on the water surface. Thin films of the above graphene composites were deposited onto different solid substrates, i.e., silicon, glass and gold-coated glass, using either electrostatic LbL or LB (LS) deposition techniques. SEM and AFM study showed that LB (or LS) films of graphene(+)CTAB had poor surface coverage and adhesion to the substrate. Electrostatic LbL deposition of graphene by alternation of graphene with oppositely charged polyelectrolytes was much more promising in these aspects. Several combinations of materials were tried including the alternation of graphene(+)CTAB and graphene(−)SDS. The best results were achieved by alternation of graphene(−)SDS with PEI. AFM study allowed the estimation the thickness of an individual graphene–surfactant flakes of about 2.0–2.5 nm.

The spectroscopic ellipsometry study of graphene thin films gave similar values for the thickness of the graphene–surfactant composite layer. While the dispersions of refractive index and extinction coefficient were modelled by a single Lorentzian peak lying in the IR region, the absorption peak of graphene at approximately 260 nm was outside the spectral range (370–1000 nm) of the ellipsometric instrument. Interesting results were obtained when studying the SPR effect in gold films coated with a few layers of graphene using both external and internal (TIRE) reflection ellipsometry. Even though graphene itself axhibits no spectral features associated with plasmon oscillations in the above spectral range, the deposition of graphene layers on gold progressively enhances the plasmon resonance in TIRE Ψ spectra and caused an extra phase shift in TIRE Δ spectra. This phenomenon can be explored in the future for enhancing the performance of SPR-based biosensors.
